# Critical Needs for Integrated Surveillance: Wastewater-Based and Clinical Epidemiology in Evolving Scenarios with Lessons Learned from SARS-CoV-2

**DOI:** 10.1007/s12560-023-09573-0

**Published:** 2024-01-02

**Authors:** Annalaura Carducci, Ileana Federigi, Giulia Lauretani, Sara Muzio, Alessandra Pagani, Nebiyu Tariku Atomsa, Marco Verani

**Affiliations:** Laboratory of Hygiene and Environmental Virology, Department of Biology, University of Pisa, Via S. Zeno 35/39, 56127 Pisa, Italy

**Keywords:** Environmental surveillance, Clinical surveillance, Public health, COVID-19, Wastewater-based epidemiology

## Abstract

During the COVID-19 pandemic, wastewater-based epidemiology (WBE) and clinical surveillance have been used as tools for analyzing the circulation of SARS-CoV-2 in the community, but both approaches can be strongly influenced by some sources of variability. From the challenging perspective of integrating environmental and clinical data, we performed a correlation analysis between SARS-CoV-2 concentrations in raw sewage and incident COVID-19 cases in areas served by medium-size wastewater treatment plants (WWTPs) from 2021 to 2023. To this aim, both datasets were adjusted for several sources of variability: WBE data were adjusted for factors including the analytical protocol, sewage flow, and population size, while clinical data adjustments considered the demographic composition of the served population. Then, we addressed the impact on the correlation of differences among sewerage networks and variations in the frequency and type of swab tests due to changes in political and regulatory scenarios. Wastewater and clinical data were significantly correlated when restrictive containment measures and limited movements were in effect (*ρ* = 0.50) and when COVID-19 cases were confirmed exclusively through molecular testing (*ρ* = 0.49). Moreover, a positive (although weak) correlation arose for WWTPs located in densely populated areas (*ρ* = 0.37) and with shorter sewerage lengths (*ρ* = 0.28). This study provides methodological approaches for interpreting WBE and clinical surveillance data, which could also be useful for other infections. Data adjustments and evaluation of possible sources of bias need to be carefully considered from the perspective of integrated environmental and clinical surveillance of infections.

**Supplementary Information:**

The online version contains supplementary material available at 10.1007/s12560-023-09573-0.

## Introduction

Wastewater-based epidemiology (WBE) is a “water fingerprinting” approach that provides an objective assessment of public and environmental health status in real time. During the SARS-CoV-2 pandemic, it has been recognized as an effective tool for monitoring viral circulation and detecting epidemic peaks and new variants (Bivins et al., 2020). Therefore, numerous countries started to implement WBE surveillance, which was subsequently recommended by the European Commission (EU, 2021/472). In Italy, SARS-CoV-2 RNA was first detected in wastewater samples collected in December 2019 (La Rosa et al., 2021a). A national project of the Italian Center for Disease Control started in March 2021 (CCM, 2020), and it was followed by a national surveillance program from October 2021 onwards (Istituto Superiore di Sanità, ISS).

Ideally, viral RNA detection from wastewater may provide a more accurate reflection of the real scale of infection circulating throughout the community than clinical case testing (Fernandez-Cassi et al., 2021), which mostly relies on symptom presentation and contact tracing. Moreover, shedding of SARS-CoV-2 in feces precedes symptoms by approximately 6 days (Petala et al., 2022); therefore, wastewater data have been proposed for the early warning of starting or resurging epidemics (Kumar et al., 2022; Randazzo et al., 2020; Xiao et al., 2022). Other advantages of this methodology compared to clinical testing are its suitability for sampling in low-resource contexts (Shrestha et al., 2021) and its lower costs (Safford et al., 2022; Shrestha et al., 2021).

Nevertheless, the application of WBE to the surveillance and control of COVID-19 requires its representativeness toward clinical epidemiology and standardization in comparison to clinical data. Numerous studies have attempted to evaluate the correlations between environmental and clinical data, obtaining variable results. As an example, Radu et al. (2022) found that the increase in wastewater signals fits the reported clinical cases, but interestingly, when the clinical trend declined, the two datasets no longer matched. On the other hand, Reynolds et al. (2022) demonstrated that viral RNA in Dublin's wastewater perfectly reflected case-based surveillance with no lead or lag time. These inconsistencies may likely arise as a consequence of the uncertainties in the variables affecting both WBE and clinical testing (Li et al., 2021).

In the case of wastewater, data uncertainties may derive from various sources from viral excretion in feces to sewage sampling and analysis: virus shedding varies among different individuals and depends on the time from the infection (Puhach et al., 2022), whereas wastewater concentration varies depending on the length and type of sewerage (separated or combined) (Hoar et al., 2022), industrial discharges (Wade et al., 2022), human activities (Xiao et al., 2022), weather factors (Foladori et al., 2021), chemical and biological components causing decay of viral signals (Bhattacharya et al., 2023; Wade et al., 2022), and fluctuations in population size contributing to the wastewater catchment area (e.g., due to tourism) (Holm et al., 2022; Rainey et al., 2022). Moreover, sampling strategies can affect outcomes due to the nature of the timing and volume (Wade et al., 2022), sample transportation and storage can affect RNA stability (Mosscrop et al., 2022), concentration methods can have different recovery efficiencies, and PCR protocols can have different limits of detection (LODs) (Arnaout et al., 2021). To standardize these data and make them comparable, biological and chemical normalization parameters can be applied based on wastewater flow rates, the population served by the monitored sewerage, sewage concentration, method efficiency, and LODs (European Commission, 2021; Hsu et al., 2022).

On the other hand, there may be uncertainties in clinical case reports, depending on the frequency and type of swab tests (i.e., antigenic or molecular) and, in the absence of systematic surveillance, on national or local regulation changes (Xiao et al., 2022). Moreover, in the case of limited testing capacity, COVID-19 cases may not be accurately captured, leading to underreporting, and people with mild or no symptoms may not be included among the clinical case data (Schmitz et al., 2021). In some cases, data may be missing or incomplete, or reporting may be delayed (Layton et al., 2022). Moreover, clinical cases can be reported as incidence or prevalence data, thus assuming different meanings when they are compared to wastewater data (D’Aoust et al., 2021).

As indicated by the recent end of the pandemic emergency, SARS-CoV-2 infection in the population is gradually evolving toward an endemic scenario (Are et al., 2023; Cohen et al., 2022), in which clinical surveillance is probably becoming less active due to a reduction in swab testing.

It is therefore very important to be aware of the sources of uncertainties when interpreting SARS-CoV-2 clinical and wastewater data and to use caution when making inferences regarding the disease epidemiology and its impact in the community. If we were able to consider all the above-mentioned factors, it would be possible to reduce the gap between estimated and real cases. Integrated environmental and clinical surveillance (IECS) could provide the most accurate view of SARS-CoV-2 circulation and COVID-19 epidemiology, facilitating the programming and evaluation of prevention measures. To this aim, both datasets should be of good quality, the sources of uncertainty should be carefully identified, and their impact on the final results should be estimated. With this approach, the surveillance results could be adjusted and fine-tuned, reducing the potential bias coming from wastewater analysis and clinical case reports.

From the perspective of IECS, we conducted a comparative analysis of clinical and WBE surveillance data in various areas of northwestern Tuscany (Italy) over a 2-year period aimed at i) investigating some determinants of discrepancies between the two datasets and ii) exploring the effect of adjustments on their comparison and integration.

## Materials and Methods

### Wastewater Sample Collection

The present study was carried out in the context of the Italian WBE SARI project (*Sorveglianza Ambientale dei Reflui in Italia*—*Environmental surveillance of wastewater in Italy*; La Rosa et al., 2020, 2021a, b) in northwestern Tuscany (Italy). The monitoring campaign spanned from February 2021 to January 2023. This study collected 390 weekly samples from four wastewater treatment plants (WWTPs) serving areas with populations ranging from 40,000 to 110,000 inhabitants (Table 1). These samples, collected as 24-h composite samples at the entrance of each WWTP, were transported to the laboratory at 4 °C and analyzed within 48 h after sampling. Nevertheless, in the studied area, the population served by each WWTP does not correspond to a single municipality but rather encompasses fractions of different municipalities. Details of the WWTP characteristics are summarized in Table 1 separately for each WWTP.

**Table 1:** Description of WWTP characteristics: served population (inhabitants), population density in WWTP areas (inh/km^2^), length of sewer network (km), sewer network structure (separated or combined) and type of sewage (household or industrial), and percentages of people from different municipalities for each WWTP

WWTP characteristics	WWTP1	WWTP2	WWTP3	WWTP4
Served population (inhabitants)^a^	42,931	68,070	110,871	60,262^b^
Population density in WWTP basin (inh/km^2^)	2307	1644	1890	2863
Length of sewerage network (km)	145.3	312.9	386.7	167.1
Sewer network structure (%)^c^	98% separated and 2% combined	11% separated and 89% combined	100% formally separated with high volume of parasitic rainwater	100% formally separated with high volume of parasitic rainwater
Type of sewage	Mainly household (< 1% industrial sewage)	Mainly household (< 1% industrial sewage)	Mainly household (0.43% industrial sewage)	Mainly household (2.11% industrial sewage)
Served municipalities (% of the served population with confirmed clinical cases contributing to RNA viral shedding)	Pisa (63.2%) San Giuliano Terme (30.1%) Vecchiano (6.7%)	Empoli (59.9%) Vinci (15.5%) Montelupo Fiorentino (13.2%) Capraia e Limite (8.0%) Cerreto Guidi (2.3%) Montespertoli (1.2%)	Massa (60.4%) Carrara (31.3%) Montignoso (8.4%) Forte dei Marmi (0.03%)	Viareggio (99.7%); Massarosa, (0.19%) Camaiore (0.05%) Vecchiano (0.04%)

^a^Served population refers to the inhabitants whose sewage effectively flows into each WWTP

^b^WWTP4 is located in a highly touristic area and experiences a 34% population increase during the summer months (July–September) compared to the rest of the year (data from the 2019–2022 period, with a slight reduction in 2020 due to pandemic restrictions; source: Tuscany Region Database, 2019–2022)

^c^In a combined sewerage system, rainwater runoff, domestic sewage, and industrial wastewater are collected into one pipe, while in a separate sewerage system, sewage and rainwater are collected separately

### Wastewater Sample Analysis

After collection, for safety reasons, the samples were pretreated at 56 °C for 30 min as reported by Zhang et al. (2022a; b). The raw sewage samples were analyzed using the analytical protocol recommended by the SARI project surveillance network, which was modified to optimize the concentration process starting in June 2021 (La Rosa et al., 2021b).

- Method A: During the initial four months of this study (February 2021 to May 2021), the starting sample volume was 250 ml, and the recovery phase relied on biphasic separation using the WHO method for poliovirus environmental surveillance (WHO, 2003) adapted by the ISS (La Rosa et al., 2020).
- Method B: The protocol was updated in June 2021 (La Rosa et al., 2021b) following the method of Wu et al. (2020), in which 45 ml of sewage was analyzed and the recovery phase was performed through centrifugation, as described by Verani et al. (2022).

Starting in October 2021, the European Commission (European Commission, 2021) recommended the inclusion of a process control virus to assess the concentration/extraction efficiency of the method. Thus, in our study, the Mengovirus strain vMC_0_ (hereafter vMC_0_) (100 μl) was used as a process control and was added to 45 ml of each sewage sample before the concentration step. The recovery rate was computed as relative quantification, considering the Ct value of the vMC_0_ spiked sample and the Ct value of the undiluted vMC_0_ (La Rosa et al., 2021b).

The extraction of viral RNA from the concentrated samples was performed using NucliSense EasyMag (bioMérieux, Marcy l’Etoile, France). Briefly, after incubation for the lysis phase (20 min), magnetic silica beads were added to adsorb RNA, and several washes were performed to remove non-adsorbed residues. A final elution was performed with 100 µl of Tris–EDTA (TE) buffer at a pH of 8.0. After nucleic acid extraction, the One-Step PCR Inhibitor Removal Kit (Zymo Research, Irvine, CA, USA) was used to remove PCR inhibitors. An inhibition control (SARS-CoV-2 RNA; 10^3^ GC/µl) was added to the extracted sample and to deionized RNAse-free water directly into the reaction mix (La Rosa et al., 2021b). The detection of SARS-CoV-2 and vMC_0_ genomes and their quantification (genome copies, GCs) was performed by one-step RT‒qPCR according to La Rosa et al. (2021b). Briefly, RT-qPCRs were carried out in a final volume of 25 µl using AgPath-ID™ One-Step RT‒PCR Reagents (Applied Biosystems), using primers, probes, and the thermal protocol described in Table S1. The standard curve used to estimate the virus titer was obtained by serial dilution (from 1.0 × 10^1^ GC/µl to 1.0 × 10^5^ GC/µl) of a synthetic dsDNA. The acceptability of amplifications was based on two criteria: the difference between the Ct of the extracted sample and the Ct of deionized water with SARS-CoV-2 RNA inhibition control ≤ 2 (corresponding to an inhibition of the PCR less than 75%) and standard curves with a slope between − 3.1 and − 3.6 and an R^2^ equal to or greater than 0.98 (Bustin et al., 2009; La Rosa et al., 2021b). The limit of detection (LOD) of the qPCR method was calculated by preparing seven dilutions of the standard dsDNA (1, 2, 3, 4, 5, 6, 10 GC/µl), each in quintuplicate. Then, the LOD was calculated as the lowest genome concentration at which all the technical replicates were positive (LOD = 3 GCs/reaction). Overall, the sewage samples were considered acceptable if the concentration/extraction efficiency based on vMC_0_ was equal to or greater than 1%.

### Clinical Data

The clinical data were obtained from the Regional Health Agency of Tuscany (Agenzia Regionale della Sanità della Toscana, ARS) database, which provided the number of newly reported positive COVID-19 cases per day for each municipality. Although a surveillance system was in place, Italy experienced several legislative changes in terms of public health rules that affected the frequency and type of clinical testing for SARS-CoV-2 infections. For the purpose of analysis, the clinical dataset was segmented into various parts related to these changes. In particular, we defined “phase” as the interval of time corresponding to a uniform public health measure and “period” as the interval of time characterized by a similar testing method.

According to these definitions, three phases were identified as follows:

- Phase 1: From February 2021 to October 2021, restrictive measures were in place to contain and prevent the epidemiological COVID-19 emergency, including interregional travel limits and closures of schools and workplaces (Italian Ministerial Decree, n°2/2021). This decree ended in the summer of 2021 (Italian government’s decree-law, n°52/2021). In this phase, all people with symptoms and all possible contacts of symptomatic or positive subjects were tested.
- Phase 2: From October 2021 to March 2022, the Italian government required a mandatory green pass (vaccination or recovery certificate or negative COVID-19 test) for workers and for access to public transportation and recreational activities (Italian government’s decree-law, n°127/2021). Such regulation led to an increase in the number of swabs, regardless of symptoms.
- Phase 3: From March 2022 to January 2023, in Italy, the state of emergency ended, and the green pass was no longer compulsory (Italian government’s decree-law, n°24/2022). It is possible that in this phase, the easing of social restrictions might have led to a reduced number of swab tests, potentially impacting the reported positivity rate. In fact, in Italy, at the end of the state of emergency (phase 3), the number of swab tests decreased by approximately 45% compared to previous phases (phases 1 and 2) (Italian Department of Civil Protection, 2023).

According to the above-mentioned definition of “period,” two intervals were identified: until the end of 2021, the reference analytical method for official swab tests was the molecular test; then, from December 2021, following a regional and national ordinance, a rapid positive antigen test was accepted to confirm a COVID-19 case, without requiring confirmation by molecular testing. This change could have affected the positivity rate of SARS-CoV-2. Therefore, the study time frame was subdivided into two periods based on the clinical testing methods used (molecular or antigenic tests):

- Period 1: From February 2021 to 28 December 2021, only molecular tests confirmed a positive COVID-19 case (Tuscany Region Ordinance, n° 23/2020).
- Period 2: From December 2021 to January 2023, both molecular and antigenic tests confirmed a positive COVID-19 case (Tuscany Region Ordinance, n° 66/2021; Circular of the Italian Ministry of Health, n° 36254/2021).

### Data Adjustment and Normalization

Both the sewage and clinical data were adjusted to take into consideration the following uncertainty factors: the analytical protocol used, the sewage flow and the served population for environmental data, and the contribution to the WWTP inflow by various municipalities for the clinical data. Other sources of variability were identified: differences among wastewater data depending on different WWTP features and differences among clinical data according to phases and periods.

#### Wastewater Data

The quantitative viral RNA data obtained through Method A were adjusted by a correction factor as described in Verani et al. (2022). Overall, the wastewater data were then normalized using Eq. 1:

1 $${NVL}_{x}= \frac{{Conc.}_{SARS-CoV-2} \times {Fd \times 10 }^{5}}{P}$$

where NVL is the normalized viral load (GCs/day/100,000 inhabitants), x is the identification number of each WWTP (namely, 1, 2, 3, 4), *Conc.*_*SARS-CoV-2*_ is the concentration of SARS-CoV-2 obtained during monitoring (GCs/L), *Fd* is the daily wastewater flow rate of WWTPs (L/day), 10^5^ is a constant used to represent the viral load of 100,000 inhabitants, and *P* is the population served by each WWTP (number of inhabitants).

As a conventional approximation (Lee et al., 2021; Noble et al., 2010), the NVLx of samples where the SARS-CoV-2 genome was not detected were assumed to have a viral genomic concentration value equal to half of the LOD (“Wastewater sample analysis” section). NVLx were Log_10_-transformed prior to statistical analysis. To represent the time evolution of the NVL in the entire study area, the geometric mean of the four NVLx was calculated for each week, and then a three-week moving average was computed as described by Zhan et al. (2022).

#### Clinical Data

The number of new positive cases was adjusted by weighting the percentage of the served population for each municipality associated with a specific WWTP (Table 1).

The adjusted clinical cases (ACCs) for each WWTP were obtained according to Eq. 2.

2 $$ACCx\, = \,\sum\nolimits_{1}^{n} {Cl_{i} } \, \times \,Fm_{i}$$

where *x* is the identification number of each WWTP (namely, 1, 2, 3, 4), *n* is the number of municipalities served by the WWTPx (Table 1), *Cl*_*i*_ is the total number of clinical cases for each municipality *i* discharging into the WWTPx during a week (n per week), and *Fm*_*i*_ is the fraction of the population of municipality *i* truly discharging into WWTPx (%; Table 1).

To make ACCx data comparable with NVLx data (from weekly sampling), they were divided by 7 factors to obtain an average weekly ACCx. In accordance with the NVL calculations, ACCs were also Log_10_-transformed, and they were represented as previously described for the NVL.

#### Data Analysis

The Shapiro‒Wilk normality test revealed that Log_10_-transformed data were not normally distributed; thus, nonparametric statistical tests were applied. The association between clinical data and the NVL, both Log_10_-transformed, was examined using Spearman’s correlation (*ρ*). Correlation analysis was performed on the pooled clinical and environmental data (representing the entire study area) and for each WWTP independently. Statistical analyses were carried out for different time frames: (i) the entire monitoring period, (ii) each of the three phases, and (iii) each of the two periods, as in “Clinical data” section. To interpret the strength of the correlation, the Spearman correlation coefficient was categorized into five classes: between 0 and ± 0.2 (negligible), between ± 0.2 and ± 0.39 (weak), between ± 0.4 and ± 0.59 (moderate), between ± 0.6 and ± 0.79 (strong), and values exceeding 0.8 or falling below -0.8 were classified as very strong correlations (Cuevas-Ferrando et al., 2022; Stachler et al., 2018). All statistical analyses were performed using GraphPad Prism 5 software (GraphPad, USA).

## Results

### Temporal Analysis of Wastewater and Clinical Data

During the 2-year study period, a total of 390 wastewater samples were collected. The SARS-CoV-2 genome was detected in 213 samples (54.6%). Considering the WWTPs separately, WWTP1 had 58 positive samples out of 96 (60.4%), WWTP2 had 47/96 (48.9%), WWTP3 had 51/99 (51.5%), and WWTP4 had 57/99 (57.5%). The viral genomic data and the NVLs are detailed in Table 2 as pooled data and separately for each WWTP.

**Table 2:** Percentage of positive samples, viral genome concentration (GCs/l), and normalized viral load (NVL; GCs/day/100,000 inhabitants) for each WWTP and considering all the WWTPs together. The mean and standard deviation (SD) of genome concentrations and NVLx were calculated for positive samples

	Number of samples (*n*)	Positive samples (%)	Log_10_ GCs/l (Mean ± SD)	Log_10_ NVL (Mean ± SD)
WWTP1	96	60.4	4.15 ± 1.13	8.51 ± 1.25
WWTP2	96	48.9	3.73 ± 1.10	8.04 ± 1.13
WWTP3	99	51.5	4.35 ± 1.04	8.57 ± 1.04
WWTP4	99	57.5	4.11 ± 1.19	8.41 ± 1.15
Pooled data	390	54.6	4.09 ± 1.13	8.39 ± 1.16

**Fig. 1:**
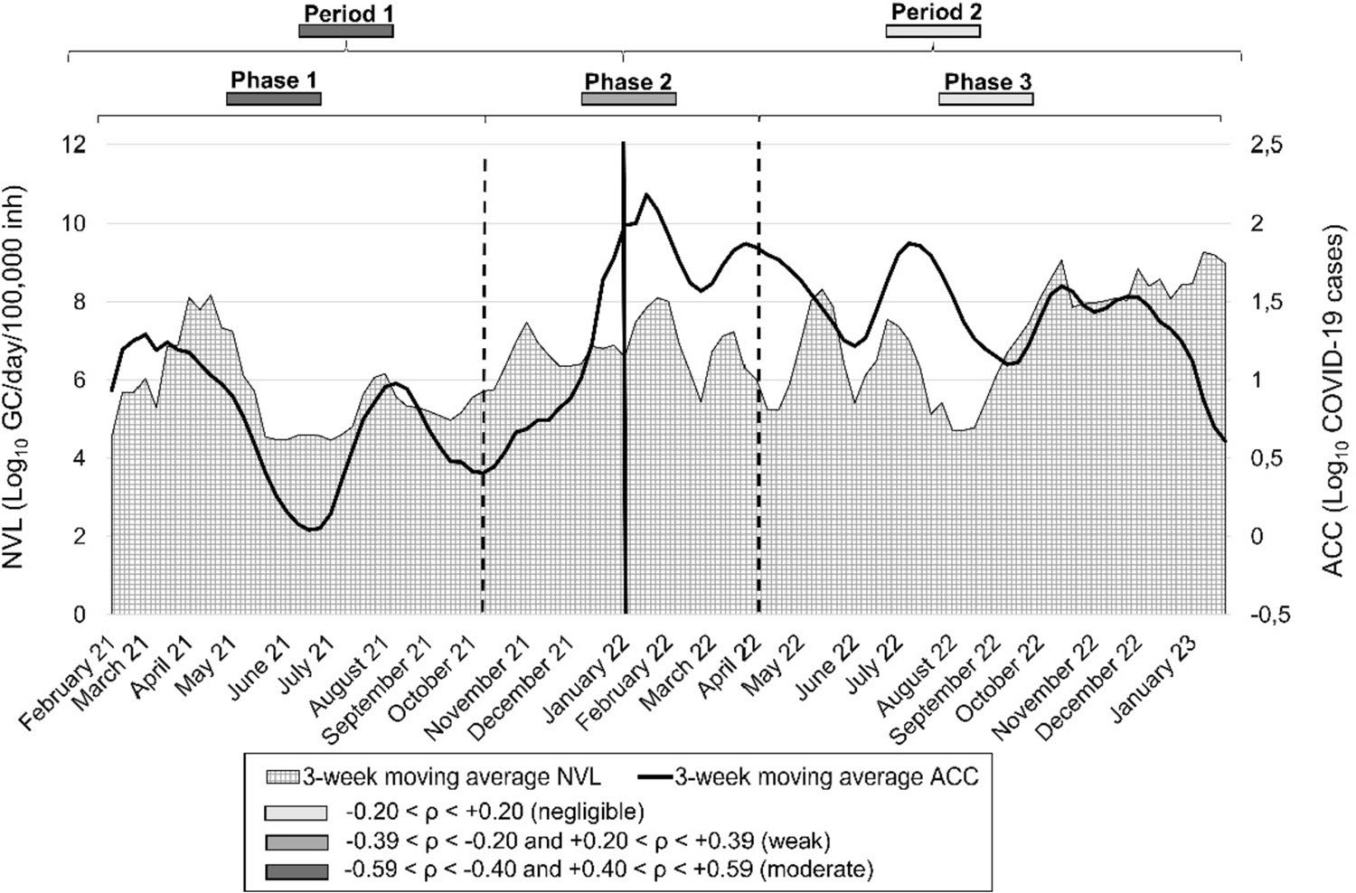
SARS-CoV-2 corrected and normalized wastewater monitoring data (light gray squared area) and COVID-19 clinical cases (continuous black line) in northwestern Tuscany from February 2021 to January 2023. *ρ* = Spearman correlation coefficient

The time trend of the NVL related to ACCs is graphically represented in Fig. 1. During period 1, when only molecular tests were considered valid for clinical confirmation of SARS-CoV-2, the registered adjusted cases followed the trend of normalized wastewater data, except for a relative peak around August. However, in period 2, a considerable increase in COVID-19 cases was observed in the entire study area.

### NVL and ACC Correlation Results

In Fig. 1, the study period was divided into different phases and periods, with the strength of the correlation between the normalized environmental (NVL) and the adjusted clinical data (ACCs). During the entire monitoring period, the association between the NVL and the ACCs of northwestern Tuscany was statistically significant. When WWTPs were considered separately, the association showed a weak positive correlation only for WWTP1 and WWTP4, *ρ* = 0.37 and *ρ* = 0.28, respectively, as reported in Fig. 1 and detailed in Table S2.

**Fig. 2:**
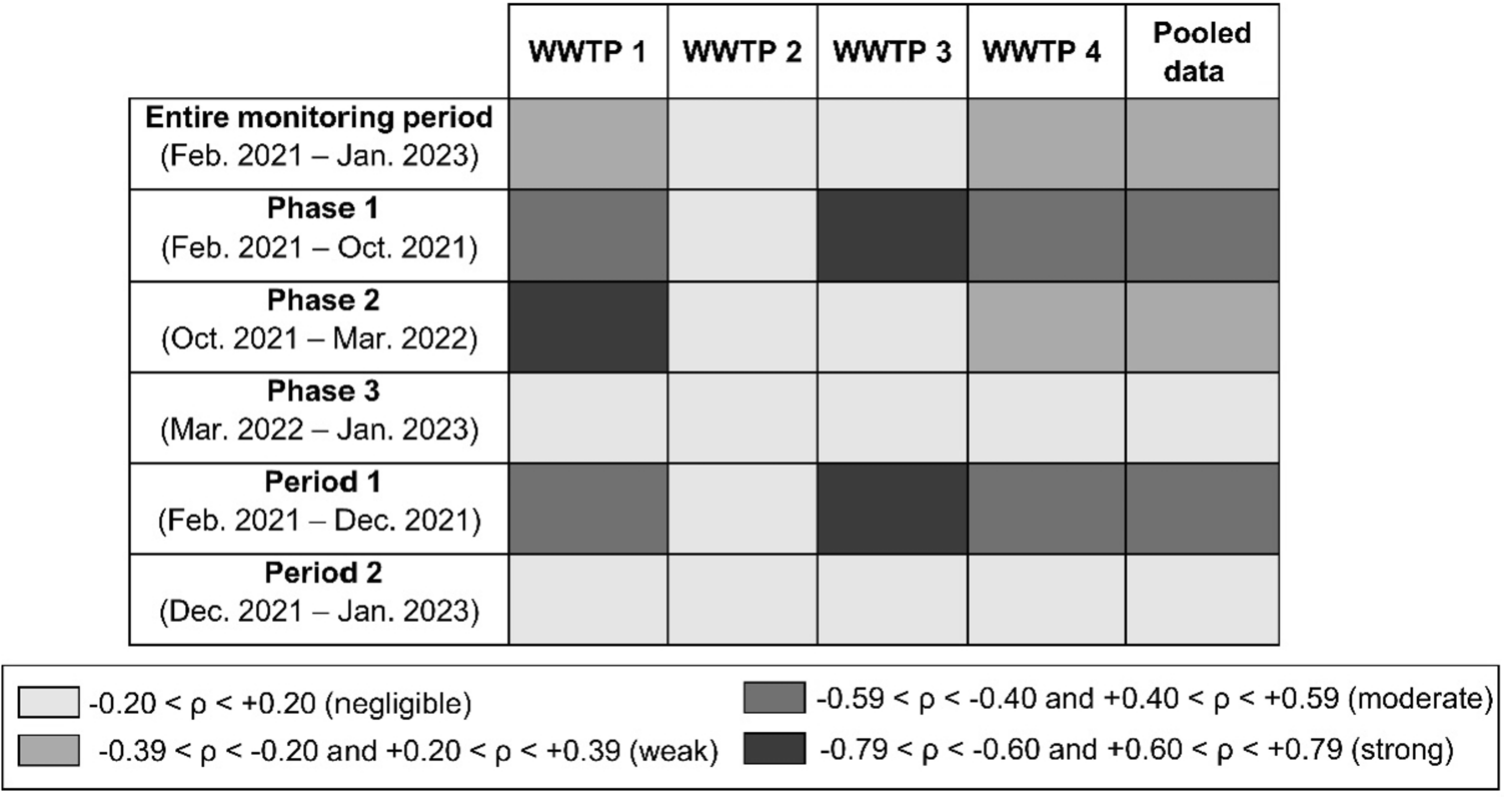
Strength of the correlation between the NVL and ACC data for the entire monitoring period (February 2021 to January 2023) and divided according to the three phases (based on the Italian government or regional policy changes) and to the two periods (based on the clinical testing method). Data are reported for each WWTP separately and as pooled data. *ρ* = Spearman correlation coefficient

Moreover, the correlation between the NVL and ACCs showed variations according to phases and periods (Fig. 2, Tables S2 and S3). In particular, considering pooled data, phases 1 and 2 had moderate and weak positive correlations, respectively (with *ρ* = 0.50 and 0.28, respectively), but this correlation was negligible in phase 3 (*ρ* = 0.02). In phase 1, WWTP1 and WWTP4 showed moderate positive correlations between the NVL and ACCs (both *ρ* = 0.49), while WWTP3 showed a strong correlation (*ρ* = 0.68), whereas in phase 2, the correlation was strong for WWTP1 (*ρ* = 0.72) and weak for WWTP4 (*ρ* = 0.35) (Table S3). When the data were divided according to the period (Fig. 2; Table S4), the NVL was positively correlated with ACCs only during period 1 (*ρ* = 0.49), where WWTP3 showed a strong correlation (*ρ* = 0.67), and WWTP1 and WWTP4 showed moderate correlations (*ρ* = 0.44 and 0.72, respectively).

## Discussion

The uncertainties in both clinical and wastewater data can be only partially reduced by applying normalization factors, as we did in the present study. In fact, in our analysis, wastewater data were adjusted on the basis of the served population and of the daily flow rate, which were identified as the most efficient correction parameters (Rainey et al., 2023). Moreover, clinical cases were adjusted taking into consideration the contribution of different municipalities to the sewerage networks. Nevertheless, these calculations were not sufficient to avoid the time and space variability of the correlation between wastewater and clinical data during a 2-year period of surveillance. In fact, in the initial phase of the study (phase 1), there was a close alignment between the two datasets considering the pooled data for the entire area and 3 out of 4 WWTPs. As the study progressed, in phase 2, this association gradually declined but in a different way among the WWTPs (e.g., it lasted longer for WWTP1), and finally, it disappeared in the last nine months of the study (Phase 3). This evolution shows the difficulty in using both WBE and clinical data to represent the real diffusion of COVID-19, especially when the infection became endemic and most cases were asymptomatic owing to vaccination.

COVID-19 clinical surveillance collects data from different sources. In this study, the daily count of new COVID-19 infections identified through swab tests was used (León et al., 2022), which is influenced by how many people in the population are being tested.

A diagnosis ratio of > 10% is considered a good indicator to correlate WBE data with clinical infections in the population (Saththasivam et al., 2021). Thus, an increase in the average daily COVID-19 tests is expected to improve the quality of the clinical and environmental data correlation. Nevertheless, the type and reliability of the tests used can influence the correlation due to differences in the sensitivity, specificity, and detection limits. Generally, antigen tests are less reliable than molecular diagnostic tests due to their lower sensitivity (Zhang et al., 2022). In fact, our study found that during period 1, when only molecular tests were used for clinical confirmation, there was a moderate positive correlation between pooled clinical and wastewater data; specifically, two WWTPs showed a moderate correlation, while one demonstrated a strong correlation. In period 2, when both molecular and antigenic tests were used, the correlation weakened or disappeared for all WWTPs.

Moreover, the frequency of COVID-19 testing is influenced by the availability of tests, the willingness of people to be tested, and the policy of testing. In our study, we identified three distinct phases: during phase 1, there were very stringent and comprehensive rules that mandated widespread clinical testing not only for symptomatic people but also for contact tracing purposes. This led to a higher detection rate of clinical SARS-CoV-2 infections, resulting in a significant correlation with the wastewater data for 3 of 4 plants. In phase 2, recent negative tests became a requirement for unvaccinated people to obtain access to work or public spaces, thus causing an increase in the number of swabs, regardless of symptoms, and then a lower positivity rate. As a result, the correlation with viral load in wastewater weakened, remaining significant only for WWTP1. In phase 3, as the state of emergency ended, testing requirements were relaxed, resulting in a substantial reduction in the number of tests and a subsequent lack of a correlation between clinical cases and viral detection in wastewater.

Additionally, the epidemiological evolution of the infection and disease due to vaccination may affect clinical case data by reducing the number of people who become symptomatic and infectious, leading to a decrease in reported cases (Swan et al., 2021), even though some studies suggest that individuals infected with SARS-CoV-2 have similar viral loads regardless of their vaccination status (Riemersma et al., 2022). Conversely, wastewater-based RNA viral signals may not reflect the same decrease in clinical cases, as the presence of viral RNA in wastewater is not necessarily a direct indicator of the number of symptomatic individuals. People who are asymptomatic or presymptomatic can still shed and excrete the virus, leading to its presence in wastewater, even if the overall confirmed clinical cases have decreased (Nourbakhsh et al., 2022). Therefore, during the first half of the study period, vaccination may not have had a major influence on the outcome of the correlation analysis, as most of the population might not have been vaccinated or had received only a limited number of vaccine doses. Hence, the viral load observed in wastewater samples likely reflected the true prevalence of infections in the community, resulting in a consistent relationship between clinical cases and viral shedding in wastewater. On the other hand, when vaccination reduced the number of clinical cases more than the prevalence of infections, the correlation between clinical and wastewater data became nonsignificant.

From these considerations, we could infer that the wastewater data provide a more reliable representation of the viral circulation in a community, while swab positivity is highly dependent on the evolution of policies. In fact, studies such as those by Xiao et al. (2022) and Zhan et al. (2022) have indicated that variations in surveillance implementation and policy changes have a greater impact on clinical data than on wastewater surveillance, especially due to the inability of clinical data to track asymptomatic cases.

Moreover, areas with denser populations tend to experience increased human activities and interactions, which can lead to higher rates of COVID-19 infections, as demonstrated in a study in southern India by Chakraborty et al. (2021) and in Algeria by Kadi and Khelfaoui (2020). This, in turn, can result in greater wastewater production and potentially higher concentrations of contaminants in the wastewater (McCall et al., 2022). While there is no conclusive evidence yet, these factors may contribute to a more robust detection of viral markers and stronger correlations between clinical and environmental data in densely populated areas, as observed in a Mumbai study by Wani et al. (2023). In the context of our study, WWTP1 and WWTP4 showed a stronger correlation between the two datasets, and in fact, they were located in areas with population densities much higher than those of WWTP2 and WWTP3.

The distance between SARS-CoV-2 sources and the WWTP is another crucial aspect to consider. As this distance increases, the wastewater has more time to undergo physical and chemical changes (Bhattacharya et al., 2023), which can affect the quality of the wastewater and the persistence of SARS-CoV-2 RNA. This may be a further explanation for the better correlation between wastewater and clinical data observed for WWTP1 and WWTP4, where the sewerage networks were markedly shorter compared to WWTP2 and WWTP3. Additionally, during transportation, the mixing of wastewater from multiple sources can lead to the dilution of the virus concentration, thus reducing its detectability: in the case of WWTP1, the sewerage network was almost completely separated, while WWTP2 had the highest percentage of combined sewerage.

To obtain a better representation of the epidemiological situation, additional information would be needed in using WBE, in particular regarding.

(I) data on hospitalizations and deaths, that would help to clarify the real extent of symptomatic cases and their relationships with WBE data, addressing the bias deriving from reduced voluntary testing;
(II) a precise definition of a “COVID-19 case” in an endemic scenario to distinguish between asymptomatic individuals with positive swab tests and those showing mild symptoms without requiring hospitalization;
(III) data on sewerage networks, for a deeper understanding of the viral signal evolution in the sewershed, especially addressing the real fraction of urban black waters.

This study has limitations due to the small size of the study area, which included four medium-sized WWTPs. Other limitations come from the incomplete identification of sources of variability. For example, the differences in wastewater data among areas indicate that they can be affected by variability deriving from various sources: external physicochemical factors such as pH, temperature, ammonia, and other chemicals in wastewater can substantially impact the detectable viral load per volume (Bertels et al., 2022). In fact, studies have shown that temperature plays a crucial role in SARS-CoV-2 viral concentration, with warmer environmental conditions leading to reduced RNA stability and faster viral decay. In fact, a recent study provided further evidence by demonstrating a significant negative correlation between temperature and the number of SARS-CoV-2 copies (Schussman et al., 2022). These findings suggest that fluctuations in environmental conditions, including temperature, could have influenced the association between wastewater data and clinical cases reported as well as variability among the four WWTPs. Nevertheless, despite these limitations, this study provides valuable insights. With enhanced normalization methods and resolution of variabilities, better correlation results may potentially be achieved and applied to broader scenarios, including larger-scale studies.

## Conclusions

Our study highlights that, when considering the combined results of wastewater RNA detection and confirmed clinical tests, there were inconsistencies throughout the pandemic and across different areas. These discrepancies can be attributed to various clinical and environmental factors that hinder the exact representation of actual cases within the population and the routine application of wastewater surveillance for public health purposes. Nevertheless, some of these discrepancies can be reduced with appropriate data adjustments. In particular, our study revealed the following:

1. Wastewater and clinical data showed a relevant correlation when both datasets were adjusted by analytical protocol and sewage flow for the WBE data and by the distribution of the population in the sewerage area for the clinical data.
2. The strength of the wastewater and clinical data correlation was influenced by other sources of bias, in particular, the characteristics of sewerage networks for the WBE data and the frequency and type of swab tests for the clinical data.

The integration of wastewater and clinical data can enhance surveillance by providing more timely information, identifying (and possibly correcting) reciprocal biases, and offering more complete information to plan and evaluate interventions. However, it is essential to exercise caution regarding the uncertainties and biases associated with each data source. From the perspective of a stable and structured integrated surveillance system for COVID-19, as well as potentially for other endemic infections, it is necessary to take into account all the influential variables for both wastewater and clinical data through the development of dedicated integrated models.

### Supplementary Information

Below is the link to the electronic supplementary material.

Supplementary file1 (DOCX 24 KB)

## Data Availability

The datasets generated during and/or analyzed during the current study are available from the corresponding author on reasonable request.
