# Supplementary material for: Critical Needs for Integrated Surveillance: Wastewater-Based and Clinical Epidemiology in Evolving Scenarios with Lessons Learned from SARS-CoV-2
Source: Food Environ Virol. 2024 Jan 2;16(1):38–49. doi: 10.1007/s12560-023-09573-0 (PMC10963525; doi:10.1007/s12560-023-09573-0)
Supplement: Supplementary file 1 — Supplementary file1 (DOCX 24 KB) [file 12560_2023_9573_MOESM1_ESM.docx]

**Supplementary Materials**

CRITICAL NEEDS FOR INTEGRATED SURVEILLANCE: WASTEWATER-BASED AND CLINICAL EPIDEMIOLOGY IN EVOLVING SCENARIOS WITH LESSON LEARNT FROM SARS-CoV-2

Annalaura Carducci^a^, Ileana Federigi^a^*, Giulia Lauretani^a^, Sara Muzio^a^, Alessandra Pagani^a^, Nebiyu Tariku Atomsa^a^, Marco Verani^a^

**^a^** Laboratory of Hygiene and Environmental Virology, Department of Biology, University of Pisa, Via S. Zeno 35/39, 56127 Pisa, Italy.

* Corresponding Author: Ileana Federigi. Laboratory of Hygiene and Environmental Virology, Department of Biology, University of Pisa, Via S. Zeno 35/39, 56127 Pisa; ileana.federigi@unipi.it

**Table of contents**

**Table S1.** Primers, probes, and amplification protocol according to Italian WBE surveillance - *SARI project* (La Rosa et al., 2021b) ………………………………………………………………………………………..2

**Table S2**. Correlation between NVL and ACC data for the entire monitoring period (February 2021 to January 2023) considering each WWTPs separately and all the data together (statistically significant results are in bold)……………………………………………………………………………………………………………2

**Table S3**. Correlation between NVL and ACC data for the three Phases divided on the Italian government or regional policy changes, considering each WWTPs separately and all the data together (statistically significant results are in bold)………………………………………………………………………………………………3

**Table S4**. Correlation between NVL and ACC data for the two Periods based on the clinical testing methods, namely molecular and antigenic tests, considering each WWTPs separately and all the data together (statistically significant results are in bold)…………………………………………………………………….3

**Table S1**. Primers, probes, and amplification protocol according to Italian WBE surveillance - *SARI project* (La Rosa et al., 2021b)

| Virus | Target region (amplicon size) | Primers and probes name | Concentrations (μM) | Sequences (5′-3′) | Thermal conditions |
| --- | --- | --- | --- | --- | --- |
| SARS-CoV-2 | ORF1ab region: nsp14; 3’-to-5’ exonuclease (100 bp) | 2297 CoV-2-F | 0.5 | ACA TGG CTT TGA GTT GAC ATC T | 50°C: 30 min,  95 °C: 5 min,  45 cycles (95 °C: 15 s; 60°C: 45 s) |
|  |  | 2298 CoV-2-R | 0.9 | AGC AGT GGA AAA GCAT GTG G |  |
|  |  | 2299 CoV-2-P | 0.25 | FAM—CAT AGA CAA CAG GTG CGC TC-MGBEQ |  |
| Mengovirus (vMC0) | 5’ untranslated regions, 5’ UTR (100 bp) | Mengo 110 (FW) | 0.5 | GCG GGT CCT GCC GAA AGT |  |
|  |  | Mengo 209 (REV) | 0.9 | GAA GTA ACA TAT AGA CAG ACG CAC AC |  |
|  |  | Mengo 147 (PROBE) | 0.2 | FAM—ATC ACA TTA CTG GCC GAA GC- MGBNFQ |  |

**Table S2**. Correlation between NVL and ACC data for the entire monitoring period (February 2021 to January 2023) considering each WWTPs separately and all the data together (statistically significant results are in bold).

|  | Number of samples (n) | Spearmann correlation coefficient (ρ) | p-value |
| --- | --- | --- | --- |
| WWTP1 | 96 | 0.37 | **< 0.001** |
| WWTP2 | 96 | 0.09 | 0.34 |
| WWTP3 | 99 | 0.06 | 0.57 |
| WWTP4 | 99 | 0.28 | **< 0.01** |
| Pooled data | 390 | 0.23 | **< 0.0001** |

**Table S3**. Correlation between NVL and ACC data for the three Phases divided on the Italian government or regional policy changes, considering each WWTPs separately and all the data together (statistically significant results are in bold)

|  | Number of samples (n) | Spearmann correlation coefficient (ρ) | p-value |
| --- | --- | --- | --- |
| **Phase 1** |  |  |  |
| WWTP1 | 34 | 0.49 | **< 0.01** |
| WWTP2 | 34 | 0.15 | 0.37 |
| WWTP3 | 32 | 0.68 | **< 0.0001** |
| WWTP4 | 32 | 0.49 | **< 0.01** |
| Pooled data | 132 | 0.50 | **< 0.0001** |
| **Phase 2** |  |  |  |
| WWTP1 | 21 | 0.72 | **< 0.001** |
| WWTP2 | 21 | 0.06 | 0.77 |
| WWTP3 | 24 | -0.05 | 0.79 |
| WWTP4 | 24 | 0.35 | 0.09 |
| Pooled data | 90 | 0.28 | **< 0.01** |
| **Phase 3** |  |  |  |
| WWTP1 | 41 | -0.14 | 0.35 |
| WWTP2 | 41 | -0.16 | 0.29 |
| WWTP3 | 43 | -0.03 | 0.84 |
| WWTP4 | 43 | 0.01 | 0.93 |
| Pooled data | 168 | 0.02 | 0.78 |

**Table S4**. Correlation between NVL and ACC data for the two Periods based on the clinical testing methods, namely molecular and antigenic tests, considering each WWTPs separately and all the data together (statistically significant results are in bold).

|  | Number of samples (n) | Spearmann correlation coefficient (ρ) | p-value |
| --- | --- | --- | --- |
| **Period 1** |  |  |  |
| WWTP1 | 44 | 0.44 | **< 0.01** |
| WWTP2 | 44 | 0.15 | 0.32 |
| WWTP3 | 42 | 0.67 | **< 0.0001** |
| WWTP4 | 42 | 0.57 | **< 0.001** |
| Pooled data | 172 | 0.49 | **< 0.0001** |
| **Period 2** |  |  |  |
| WWTP1 | 52 | -0.03 | 0.78 |
| WWTP2 | 52 | -0.09 | 0.50 |
| WWTP3 | 57 | -0.05 | 0.69 |
| WWTP4 | 57 | -0.00 | 0.98 |
| Pooled data | 218 | 0.03 | 0.57 |
